# Antimicrobial effect of methylene blue in microbiologic culture to diagnose periprosthetic joint infection: an in vitro study

**DOI:** 10.1186/s13018-022-03475-w

**Published:** 2022-12-28

**Authors:** Kan Liu, Yanping Luo, Libo Hao, Jiying Chen

**Affiliations:** 1 Department of Orthopedics, Beijing University of Chinese Medicine Third Affiliated Hospital, No.51 Xiaoguan Street, Beijing, 100029 China; 2 Department of Clinical Microbiology, General Hospital of Chinese People’s Liberation Army, Beijing, China; 3 Department of Orthopedics, General Hospital of Chinese People’s Liberation Army, Beijing, China

**Keywords:** Methylene blue, Periprosthetic joint infection, Periprosthetic tissue culture, Biofilm, Antibacterial effect

## Abstract

**Background:**

As one of the major diagnostic criteria in Musculoskeletal Infection Society, the microbiological diagnosis of periprosthetic joint infection (PJI) performed by analyzing periprosthetic tissue culture is recommended. The goal of this study was to determine if methylene blue (MB) has antibacterial effects that might interfere with microbial culture in vitro.

**Methods:**

Eight isolates of reference strains of *Staphylococcus aureus, Staphylococcus epidermidis, Staphylococcus hominis, Escherichia coli, Klebsiella pneumoniae, Acinetobacter baumannii, Streptococcus pyogenes,* and *Candida albicans* were incubated appropriately on blood agar, China blue agar, or Sabouraud’s agar plates at 35 ℃. (*Streptococci* were cultured in a CO_2_-rich atmosphere.) Each bacterial suspension was formed by 50-fold dilution before the test MB was added. For each strain, bacterial suspension was divided into 3 groups (5 samples each) exposed either MB 0.1%, MB 0.05% or sterile non-bacteriostatic 0.45% saline. The antimicrobial property of MB was determined by measuring the bacterial density on agar plates incubated for 24 h and comparing it with controls unexposed to MB.

**Results:**

Exposure to MB 0.1% or MB 0.05% negatively affected microbial viability in vitro. Of the diluted form of MB exposure, reference strains of *S. hominis* and *A. baumannii* resulted in fewer colony-forming units compared with the sterile saline control. MB concentration was significantly negatively correlated with CFU counts of *S. hominis* and *A. baumannii* strains. The antibacterial property of MB 0.1% or MB 0.05% appears to affect the ability to culture the organism in in vitro assays.

**Conclusion:**

MB 0.1% or MB 0.05% has strong antimicrobial activities against some commonly encountered bacterial strains in PJI in vitro. To further evaluate its potential antibacterial usefulness in clinical applications, the next studies are needed to assess the ability of MB to affect the ability to culture the pathogens in vivo, especially in periprosthetic tissue.

## Background

Periprosthetic joint infection (PJI) is a catastrophic complication following total joint arthroplasty (TJA) [1], which accounts for 1–2% of hip arthroplasties and knee arthroplasties [2, 3]. In the management of PJI, the ability to identify septic and aseptic failures of the prosthesis would be critical for the surgeon in deciding on optimal treatment of PJI [4]. Notably, isolation of bacteria from periprosthetic tissue or synovial fluid is essential to determine antimicrobial susceptibilities of the organisms.

The microbiological analysis of periprosthetic tissue and synovial fluid, the hematology, and imaging tests are important routines for diagnosing PJI, but some of these results are nonspecific and nonsensitive for PJI. To address the inconsistency of different tests for diagnosing PJI, in 2018, the modified Musculoskeletal Infection Society (MSIS) renewed this with a consensus statement providing a concise definition of PJI [5]. As one of the major diagnostic criteria in MSIS, the microbiological diagnosis of PJI performed by analyzing periprosthetic tissue culture or synovial fluid culture is recommended. However, until now, the definitive diagnosis of PJI is still difficult and is mainly characterized by technical limitations and unsatisfactory results of culture.

The presence of bacterial biofilm has been a topic of concern for diagnosis and eradication of implant-related bone and joint infections [6]. PJI is associated with biofilm formation on the implant surface where the bacteria with substantial protection against antimicrobial agents and the host immune response pose a major challenge to diagnose and eradicate [7–9]. As such, the ability to identify biofilms intraoperatively would be useful in the management of PJI. Identifying biofilms may help the surgeon decide on optimal management of the acute PJI. Furthermore, if a 2-stage exchange arthroplasty is needed, the ability to identify biofilms on the cement spacer intraoperatively before reimplantation could be advantageous [10].

According to the results of recent studies, several disclosing agents with various contents may have a role in quickly detecting biofilms on orthopedic prostheses [9–12]. To be used effectively in an operative setting, a disclosing agent would need to stain biofilm on the implant or tissue and have limited antimicrobial activity, so the organism could be grown in culture. However, safranin, crystal violet, eosin, and erythrosine dyes are all disclosing agents that stain biofilm but are limited by substantial antimicrobial activity [10]. Thus, their main effects are used in microbiological sampling can pose a problem by causing false-negative results.

As a disclosing agent, methylene blue (MB) is a phenothiazine dye with biofilm-staining capabilities but also has known antimicrobial actions that are enhanced by the absorption of light, with antibacterial action mediated via DNA damage, while remaining nontoxic in humans [13]. Based on reports, the diluted form of MB does not appear to substantially affect microbial activity after staining [8, 10]. Based on the literature, we hypothesized that the results of microbial culture are adversely affected by use of MB for sampling. To date, there are uncertain findings of whether MB affects the ability to culture the organism in diagnosing PJI. Thus, we designed an in vitro study to mimic the clinical situation with regard to isolates exposed to the diluted solutions of MB. The purpose of this study was to determine if the diluted form of MB interferes with the ability to culture the pathogenic bacteria commonly encountered in PJI.

## Materials and methods

To preliminarily examine the concept that methylene blue (MB) can produce false-negative results of bacterial culture, an in vitro experiment was performed to test the antibacterial properties of MB on common PJI pathogenic bacteria, which was the same as the previous publication of our study [12]. The bacteria used in this study were 8 isolates of reference strains of *Staphylococcus aureus* (SAU) (ATCC 25923), *Staphylococcus epidermidis* (SEP) (ATCC 14990), *Staphylococcus hominis* (SHO) (ATCC 27844), *Escherichia coli* (ECO) (ATCC 11775), *Klebsiella pneumoniae* (KPN) (ATCC 13883), *Acinetobacter baumannii* (ABA) (ATCC 19606), *Streptococcus pyogenes* (SPY) (ATCC 12344), and *Candida albicans* (CAL) (ATCC 90028), all originally from the American Type Culture Collection, Manassas, VA). The experiment quantified the amount of bacteria on agar plates. Colony-forming units (CFU) were counted, and results are expressed as CFU/ml for samples.

We performed tenfold, 25-fold, 50-fold, and 100-fold dilutions and were quantitatively cultured 10 μl of the resultant fluids by plating onto Mueller–Hinton (MH) agar (bioMerieux) plates to estimate the concentration before the test MB was added. As a result, we prepared an optimal inoculum size of 3 × 10^6^ colony-forming units (CFU)/ml. This was achieved by culturing the bacteria overnight on blood agar, China blue agar, and Sabouraud’s agar plates at 35 ℃ (*Streptococci* were cultured in a CO_2_-rich atmosphere). All cultures were then diluted with sterile non-bacteriostatic 0.45% saline to achieve a 0.5 McFarland standard approximately 1.5 × 10^8^ CFU/ml, which were then diluted to reach a final concentration of approximately 3 × 10^6^ CFU/ml. This was achieved by diluting 100 μl of the 0.5 McFarland standard with 4.9 ml of sterile saline by using a sterile pipette and its tip.

Quantitative culture was performed on each bacterial suspension to which MB 0.1% or MB 0.05% was added. In addition, as a control group, a sample of each bacterial suspension was unexposed to MB and was plated onto MH agar (bioMerieux) and MH agar with 5% sheep blood (bioMerieux) (for SPY) plates for 24 h at 35 ℃ (SPY in CO_2_-rich atmosphere). The sample of each bacterial suspension in either exposed groups or unexposed groups was incubated on 5 agar plates. All quantitative cultures were performed on each agar in triplicate.

MB (Jumpcan Pharmaceutical Group Co., Ltd. 2 ml: 20 mg) with concentrations of 0.1% and 0.05% was chosen to simulate clinical practice and circumstance in our center. Tests with MB were prepared by modifying previously described methods, which were diluted by 2 ml 1% MB/18 ml normal saline and 2 ml 1% MB/38 ml normal saline [10–12]. Initially, 3 polystyrene tubes for each isolate were prepared, one containing 100 μl MB 0.1% and 600 μl bacterial suspension (3 × 10^6^ CFU/ml), one containing 100 μl MB 0.05% and 600 μl bacterial suspension (3 × 10^6^ CFU/ml), and the other containing 100 μl sterile saline and 600 μl bacterial suspension (3 × 10^6^ CFU/ml). Then, the tubes were mixed for 1 min and the resultant suspensions of 10 μl were inoculated onto MH agar plates and incubated for 24 h at 35 ℃ (SPY in a CO_2_-rich atmosphere). After incubating on the agar plates for 24 h, colonies were counted by 2 independent researchers.

## Statistical analysis

SPSS 19.0 software (IBM, Chicago, IL) was used to compare data among groups. Data are presented as mean ± standard deviation for continuous variables. These data included in vitro CFU counts of the MB group and control group, compared using the *t* test or the one-way ANOVA. Spearman correlation analysis was performed between MB concentration and in vitro CFU counts. Values of *P* < 0.05 were considered significant for all statistical comparisons.

## Results

**Fig. 1:**
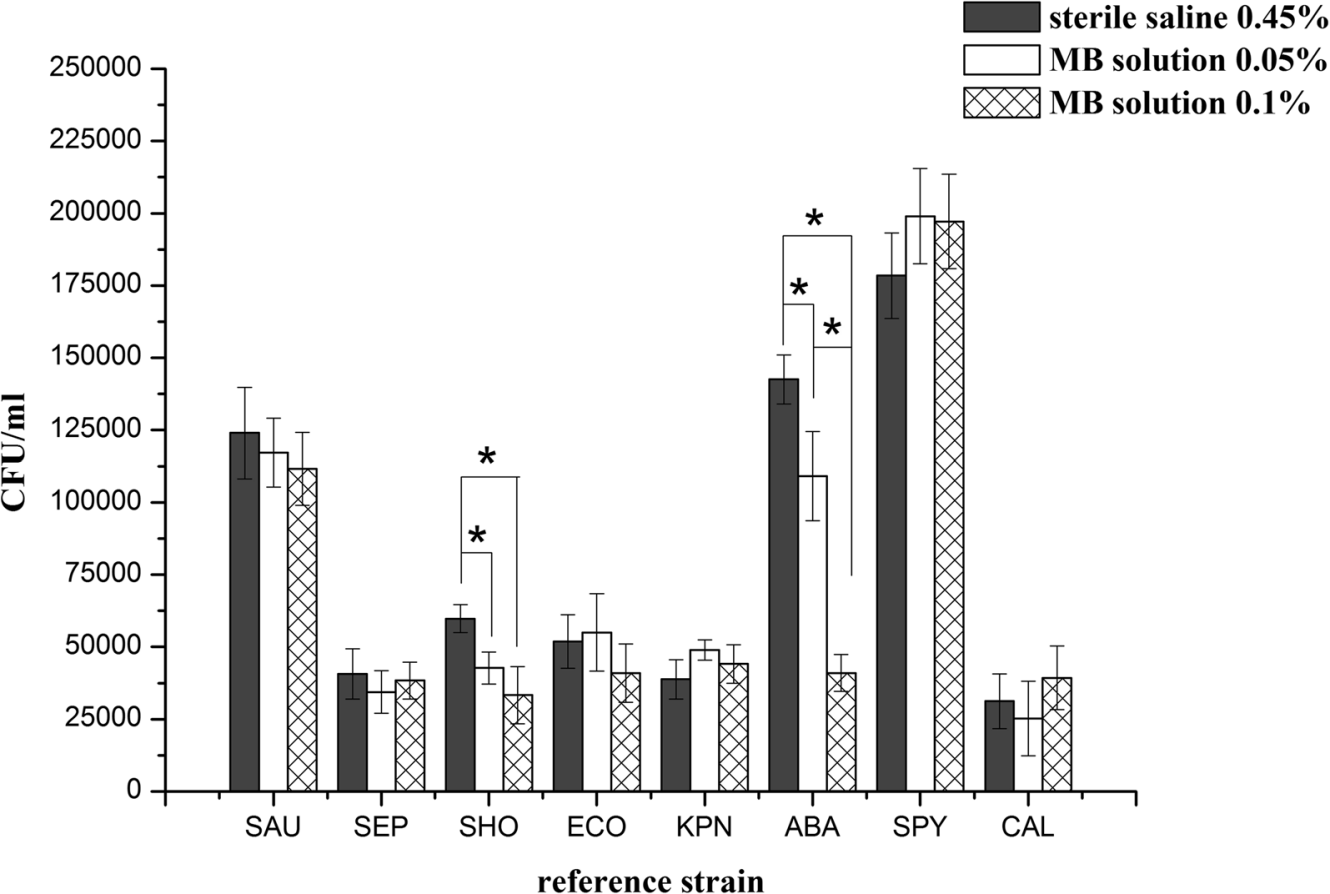
The mean density of bacteria incubated on agar plates was quantified after 24 h (and standard deviation). *P < 0.05 for the differences among MB 0.1%, MB 0.05% and sterile saline 0.45% groups as shown by the t test or the one-way ANOVA. MB= methylene blue, CFU = colony-forming unit, SAU = *Staphylococcus aureus*, SEP = *Staphylococcus epidermidis*, SHO = *Staphylococcus hominis*, ECO = *Escherichia coli*, KPN = *Klebsiella pneumoniae*, ABA = *Acinetobacter baumannii*, SPY = *Streptococcus pyogenes*, CAL = *Candida albicans*

Quantitative culture of ATCC strains quantified the amount of bacteria on agar plates. Exposure to MB 0.1% and MB 0.05% decreased microbial viability for SHO and ABA strains. With regard to MB 0.1% and MB 0.05%, a test with sterile saline as a growth control showed no antibacterial effect (Fig. 1). Quantitative culture of SAU, SEP, SHO, ECO, KPN, ABA, SPY, and CAL exposed to MB 0.1% estimated a mean bacterial density (± standard deviation) of (11.16 ± 1.26) × 10^4^, (3.84 ± 0.64) × 10^4^, (3.33 ± 0.99) × 10^4^, (4.10 ± 1.01) × 10^4^, (4.41 ± 0.66) × 10^4^, (4.10 ± 0.64) × 10^4^, (19.72 ± 1.64) × 10^4^, and (3.93 ± 1.10) × 10^4^ CFU/ml, respectively. As the same, quantitative culture of the above strains exposed to MB 0.05% estimated a mean bacterial density of (11.72 ± 1.19) × 10^4^, (3.44 ± 0.74) × 10^4^, (4.27 ± 0.56) × 10^4^, (5.50 ± 1.34) × 10^4^, (4.90 ± 0.35) × 10^4^, (10.91 ± 1.54) × 10^4^, (19.90 ± 1.65) × 10^4^, and (2.52 ± 1.29) × 10^4^ CFU/ml, respectively. Mean bacterial density on agar plates in the unexposed controls was (12.40 ± 1.58) × 10^4^, (4.07 ± 0.87) × 10^4^, (5.98 ± 0.48) × 10^4^, (5.19 ± 0.93) × 10^4^, (3.88 ± 0.68) × 10^4^, (14.25 ± 0.85) × 10^4^, (17.84 ± 1.48) × 10^4^, and (3.12 ± 0.95) × 10^4^ CFU/ml, respectively (Table 1).

**Table 1:** Bacterial density on agar plates in exposed groups and control groups (data are mean ± standard deviation) (× 10^4^ CFU/ml)

Reference strain	MB 0.1%	MB 0.05%	Sterile saline 0.45%	*P* value
SAU	11.16 ± 1.26	11.72 ± 1.19	12.40 ± 1.58	0.3795
SEP	3.84 ± 0.64	3.44 ± 0.74	4.07 ± 0.87	0.4363
SHO	3.33 ± 0.99	4.27 ± 0.56	5.98 ± 0.48	< 0.001#
ECO	4.10 ± 1.01	5.50 ± 1.34	5.19 ± 0.93	0.1531
KPN	4.41 ± 0.66	4.90 ± 0.35	3.88 ± 0.68	0.0519
ABA	4.10 ± 0.64	10.91 ± 1.54	14.25 ± 0.85	< 0.001#
SPY	19.72 ± 1.64	19.90 ± 1.65	17.84 ± 1.48	0.1179
CAL	3.93 ± 1.10	2.52 ± 1.29	3.12 ± 0.95	0.1795

*MB* methylene blue, *CFU* colony-forming unit, *SAU* *Staphylococcus aureus*, *SEP* *Staphylococcus epidermidis*, *SHO* *Staphylococcus hominis*, *ECO* *Escherichia coli*, *KPN* *Klebsiella pneumoniae*, *ABA* *Acinetobacter baumannii*, *SPY* *Streptococcus pyogenes*, *CAL* *Candida albicans*

#Significant

When exposed to MB 0.1% and MB 0.05%, there were a very rapid and significant reduction of SHO and ABA strains within 24 h compared with saline controls (*P* < 0.05) (Fig. 1), while SAU, SEP, ECO, KPN, SPY, and CAL were less susceptible when exposed to whether MB 0.1% and MB 0.05% after 24 h, with no significant reduction shown on the plates (*P* > 0.05). Spearman correlation analysis of MB concentration and CFU counts is shown in Table 2. MB concentration was significantly negatively correlated with CFU counts of SHO and ABA strains (*P* < 0.05).

**Table 2:** Spearman correlation analysis of MB concentration and CFU counts of different strains

Reference strain	*r* value	*P* value
SAU	− 0.340	0.215
SEP	− 0.094	0.738
SHO	− 0.832	< 0.001#
ECO	− 0.454	0.089
KPN	0.416	0.123
ABA	− 0.945	< 0.001#
SPY	0.472	0.075
CAL	0.283	0.306

*MB* methylene blue, *CFU* Colony-forming unit, *SAU Staphylococcus aureus*, *SEP Staphylococcus epidermidis*, *SHO Staphylococcus hominis*, *ECO Escherichia coli*, *KPN Klebsiella pneumoniae*, *ABA Acinetobacter baumannii*, *SPY Streptococcus pyogenes*, *CAL Candida albicans*

#Significant

## Discussion

Orthopedic surgeons use MB in multiple applications. It can be used to provide a visual index of surgical debridement in the treatment of PJI [9, 13]. It can also be added to bone cement in total joint arthroplasty, as an adjunct to assess traumatic arthrotomy, and also to demarcate sinus tracts for excision in the setting of PJI [11, 15, 16]. MB is a phenothiazine dye that possesses antimicrobial properties [8, 10, 14]. Earlier study has demonstrated that the antibacterial effect of MB is related to induced DNA damage in bacteria when exposed to light, while remaining nontoxic in humans in topical administration [14]. Results in the present study showed that from MB 0.1% and MB 0.05% exposure, reference strains only SHO and ABA strains resulted in less colony-forming units compared to the sterile control. We concluded that the diluted MB solution has certain antimicrobial activities against some commonly encountered bacterial strains in PJI in vitro.

Isolation of bacteria from periprosthetic tissue or synovial fluid is essential to diagnose PJI and determine antimicrobial susceptibilities of the organisms. Silva et al. [17] compared the plaque-staining ability and antimicrobial properties of various stains, including 0.05% MB, 4% erythrosine, and 1% neutral red, and found that the diluted form of MB had the best staining properties and limited antimicrobial effects. Parry et al. [10] reported that MB is a possible cost-effective and novel method to expeditiously identify intraoperative biofilm, and staining did not affect the ability to culture the organism. Taken together, the diluted MB solution appears to have limited antimicrobial properties. Based on these studies, our experiment also used MB in diluted form, applied by intraoperative staining. Our purpose was not to show the possibility of using MB as an antibiotic but, rather, to test whether it had any antimicrobial effects and provided false-negative results in microbial culture for diagnosing PJI.

With MB 0.1% or MB 0.05% versus sterile saline controls, we found no significant reduction of bacterial density on MH agar plates incubated at 35 ℃ for 24 h, except for reference strains of SHO and ABA, which were decreased after 24 h of incubation. Also, MB was shown to have concentration-dependent antibacterial activity in our study. The diluted MB solution appears to have certain antimicrobial effects. Our results are consistent with an oft-cited study by Briggs et al. [8], who found *Staphylococci* were eradicated at the lowest concentration of 0.1 mM MB. With *A. baumannii,* increasing the MB concentration improved the bactericidal effect. However, in contrast to our study, Shaw et al. [11] found more bacteria were found in MB stained vs unstained tissue-based on semiquantitative culture. Moreover**,** an in vitro experiment confirmed that exposure to MB 0.05% did not negatively affect microbial viability [10]. As far as the above results concerned, we think the properties of MB and bacterial, experimental methods, culture media, and culture conditions may have contributed to the differences. Therefore, more research evidence is needed to confirm the antimicrobial properties and antimicrobial spectrum of MB.

As mentioned above, the results of bacterial reduction found at 37 ℃ [8] were almost the same as we found at 35 ℃. Therefore, temperature does not seem to affect the antimicrobial property of MB. Besides, the present study did attempt to test whether antimicrobial effect varied with different concentrations, so two different concentrations of MB used routinely in our department were chosen in order to mimic clinical practice of periprosthetic tissue sampling. MB, a well-known staining dye for using to highlight dental plaque [18], has recently shown promise as a biofilm-disclosing agent in 2 pilot studies in the arthroplasty literature [10, 11], may have antimicrobial activity with the use of its routine concentration, as in our study. Thus, as MB has mixed effects on different bacterial strains, the diluted form of MB may be inappropriate for the preparation of periprosthetic tissue sampling, even though the additive-free solution may potentially identify biofilm. From the aforementioned findings, the warnings of antibacterial activity stated in MB must be taken into consideration, especially in the preparation of periprosthetic tissue sampling.

The present study is limited by the use of MB with no attempt made to protect the specimens from light, which may lead to a negative result of culture. However, in practice, incubation did occur in the dark, which may have reduced the antimicrobial action of MB. In addition, the density of bacterial suspension in this study was supraphysiological and the intraoperative application of MB could not be completely protected from light. Thus, we do not think this limitation render our results unreasonable.

## Conclusions

In summary, the colonies of viable bacteria measured for MB 0.1% or MB 0.05% indicate the antibacterial property of this agent in vitro. As the bacterial density incubated in this study was supraphysiological, we have good reason to believe that the diluted form of MB has strong antibacterial effects against some common PJI pathogenic bacteria in in vitro assays**.** Therefore, we speculate that staining biofilm with MB 0.1% or MB 0.05% may be inappropriate due to its antimicrobial effects. On the other hand, for it to be considered for the periprosthetic tissue sampling for culture, future investigations are needed to demonstrate whether MB can affect the ability to culture the bacteria in the biofilm in vivo.

## Data Availability

Not applicable.
